# 147 Development of the Czech version of the tool for assessing physical activity policy at national and sub-national level

**DOI:** 10.1093/eurpub/ckae114.159

**Published:** 2024-09-26

**Authors:** Josef Mitáš, Michael Pratt, Andrea Ramírez-Varela, Eugen Resendiz, Juliana Mejía-Grueso, Aleš Jakubec, Deborah Salvo

**Affiliations:** Faculty of Physical Culture, Palacký University Olomouc, Olomouc, Czech Republic; Faculty of Physical Culture, Palacký University Olomouc, Olomouc, Czech Republic; Herbert Wertheim School of Public Health and Human Longevity Science, University of California San Diego, San Diego, USA; Global Observatory for Physical Activity – GoPA!; Department of Epidemiology, School of Public Health, UTHealth Science Center at Houston, Houston, USA; Center for Pediatric Population Health, School of Public Health, UTHealth Science Center at Houston, Houston, USA; Department of Pediatrics at McGovern Medical School, UTHealth Science Center at Houston, Houston, USA; Global Observatory for Physical Activity – GoPA!; Department of Kinesiology and Health Education, The University of Texas at Austin, Austin, USA; Global Observatory for Physical Activity – GoPA!; Faculty of Physical Culture, Palacký University Olomouc, Olomouc, Czech Republic; Department of Kinesiology and Health Education, The University of Texas at Austin, Austin, USA

## Abstract

**Purpose:**

This study aimed to adapt and implement the Czech version of the “Interaction between National and Local Government Levels in Development and Implementation of Physical Activity Policies Tool” (INTEGRATE-PA-Pol). This tool examines the cross-level interactions between national and sub-national government levels during the development and implementation of health-enhancing physical activity (HEPA) policies.

**Methods:**

The research was carried out in three stages: (1) translation of the English tool into Czech by three independent translators; (2) adapt the local-level sections of the six questionnaires to assess regional-level interactions; (3) conduct cognitive response testing and item modification to validate the translation in terms of Czech terminology; and (4) pilot test of the tool across national, regional and local levels of government.

**Results:**

The Czech version of the INTEGRATE PA-Pol tool evaluates the cross-level perspective of collaboration between national, regional, and local governments during the development and implementation of HEPA policies. This version was tested at the national level, regional (Olomouc), and local levels (Olomouc city). We successfully identified involved sectors and key contacts and collected data for policymakers at the national (n = 2), regional (n = 2), and local (n = 1) levels.

**Conclusions:**

The Czech adaptation of the INTEGRATE PA-Pol tool was developed to gain a deeper understanding of the design and implementation of physical activity policies at all levels of government involved in the physical activity policies in the Czech Republic. Our study showed the feasibility of adapting the tool to Czech and collecting data for the regional leveĺs involvement in HEPA policies in a European country. Our results will contribute to developing and implementing GoPA!’s global HEPA policy monitoring system. The interview process itself demonstrated important differences in prioritization for physical activity between sectors and government levels.

